# Supplementary figures and images for: An anatomically informed computational fluid dynamics modeling approach for quantifying hemodynamics in the developing heart
Source: PLoS One. 2025 May 19;20(5):e0322233. doi: 10.1371/journal.pone.0322233 (PMC12088024; doi:10.1371/journal.pone.0322233)

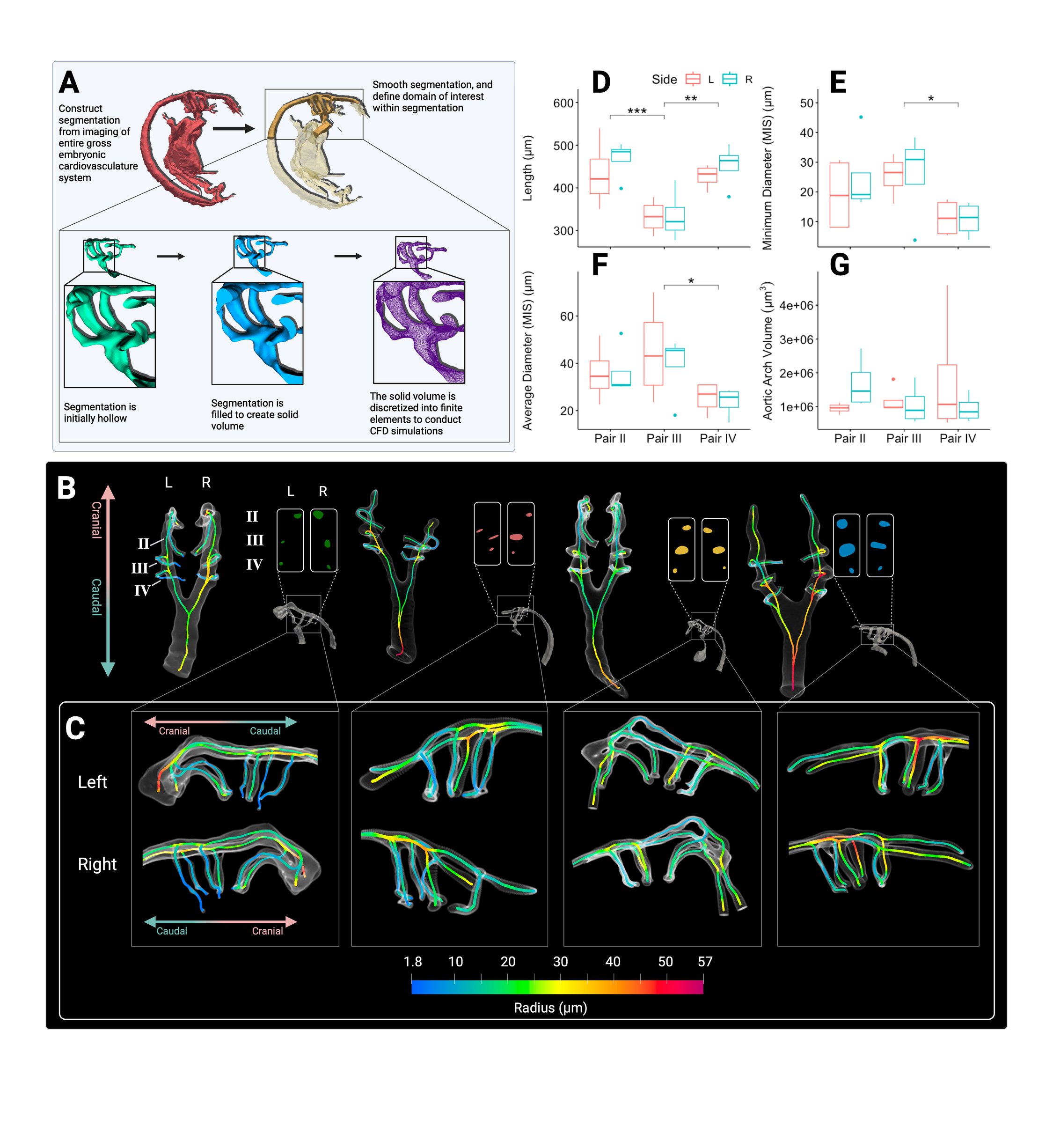

Supplement: S1 Fig — (A) Following manual segmentation from imaging, the region of interest was selected which included the outflow tract, AAs, and dorsal aorta. Next the segmentation was smoothed, solidified and discretized into a tetrahedral mesh. (B) Aortic arch cross sections are elliptical in shape at this stage. (C) AA III contains the largest average diameter, while AA IV contains the smallest minimum and average diameter. (D-G) Arches were compared laterally (left/right sides) and pairwise (AA II/AA III/ AA IV) using ANOVA followed by Tukey analysis (where * indicates P < 0.05, ** indicates P < 0.01, *** indicates P < 0.001). In general, arches varied by pair, but not laterally (left vs. right). Diameters were calculated by measuring the diameter of the maximally inscribed sphere (MIS) at a given point in the arch. (D) The central arch, AA III was significantly shorter than either of the other arches flanking it. (E) AA IV had the smallest minimum diameter among the three pairs and was significantly smaller than the minimum diameter of AA III. (F) AA IV contained the smallest average diameter and had a significantly smaller average diameter compared to AA III. (G) Aortic arch volumes did not significantly differ by side or pair. (TIF) [file pone.0322233.s001.tif]

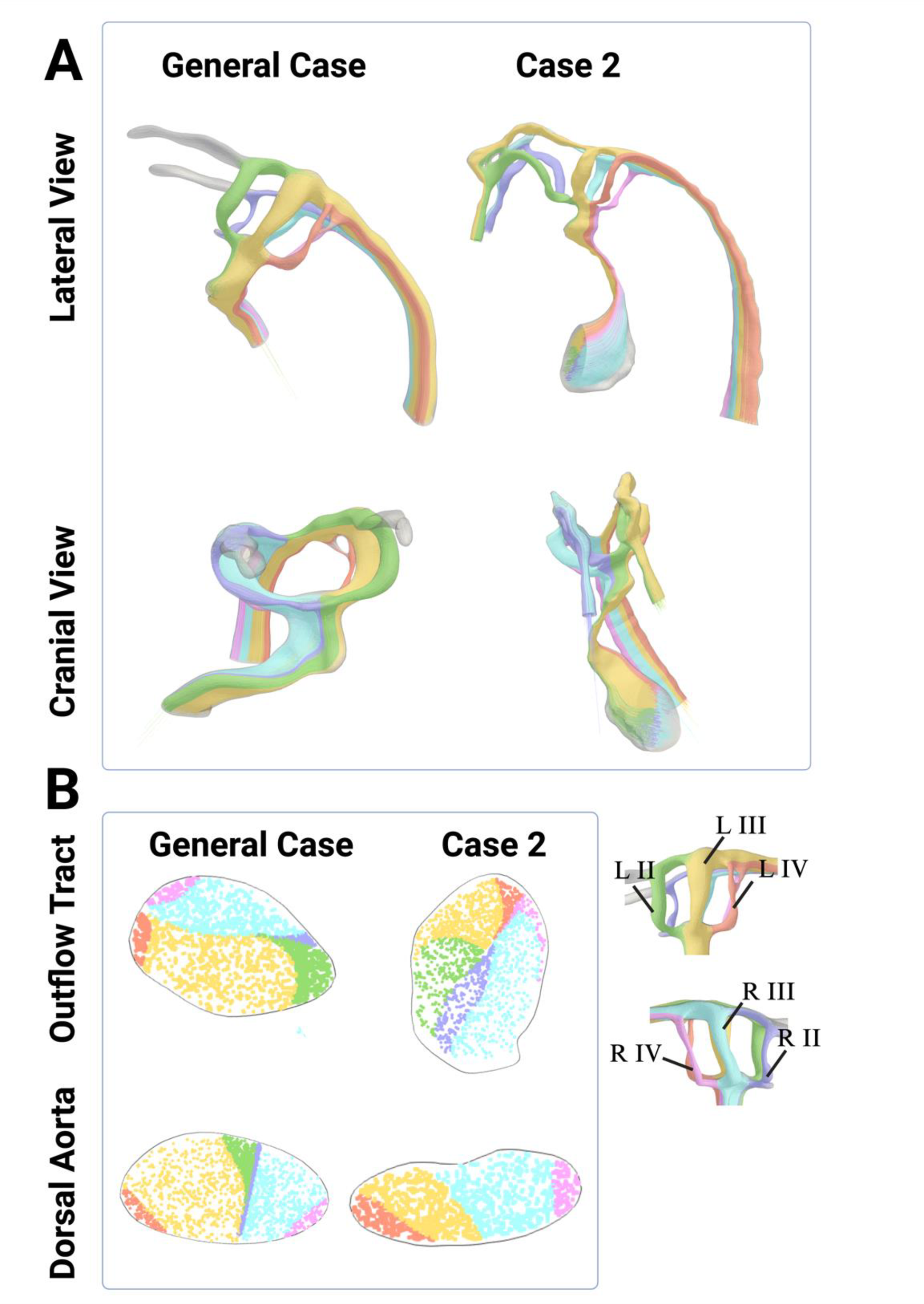

Supplement: S2 Fig — In case 2, blood flow is simulated to flow out the cranial arches in addition to the dorsal aorta. Case 2 streamlines are shown next to a general case for comparison. (A) Lateral and cranial views of case 2 compared to the general case are shown. Streamlines from R II, R III, L II, and L III can be observed exiting through the cranial arteries. (B) The cross sections of the outflow tract and dorsal aorta are shown. Streamlines for RII and L II do not exit through the dorsal aorta in case 2, however the remaining streamlines maintain the same spatial orientation compared to the general case in the absence of L II and R II streamlines. (TIF) [file pone.0322233.s002.tif]

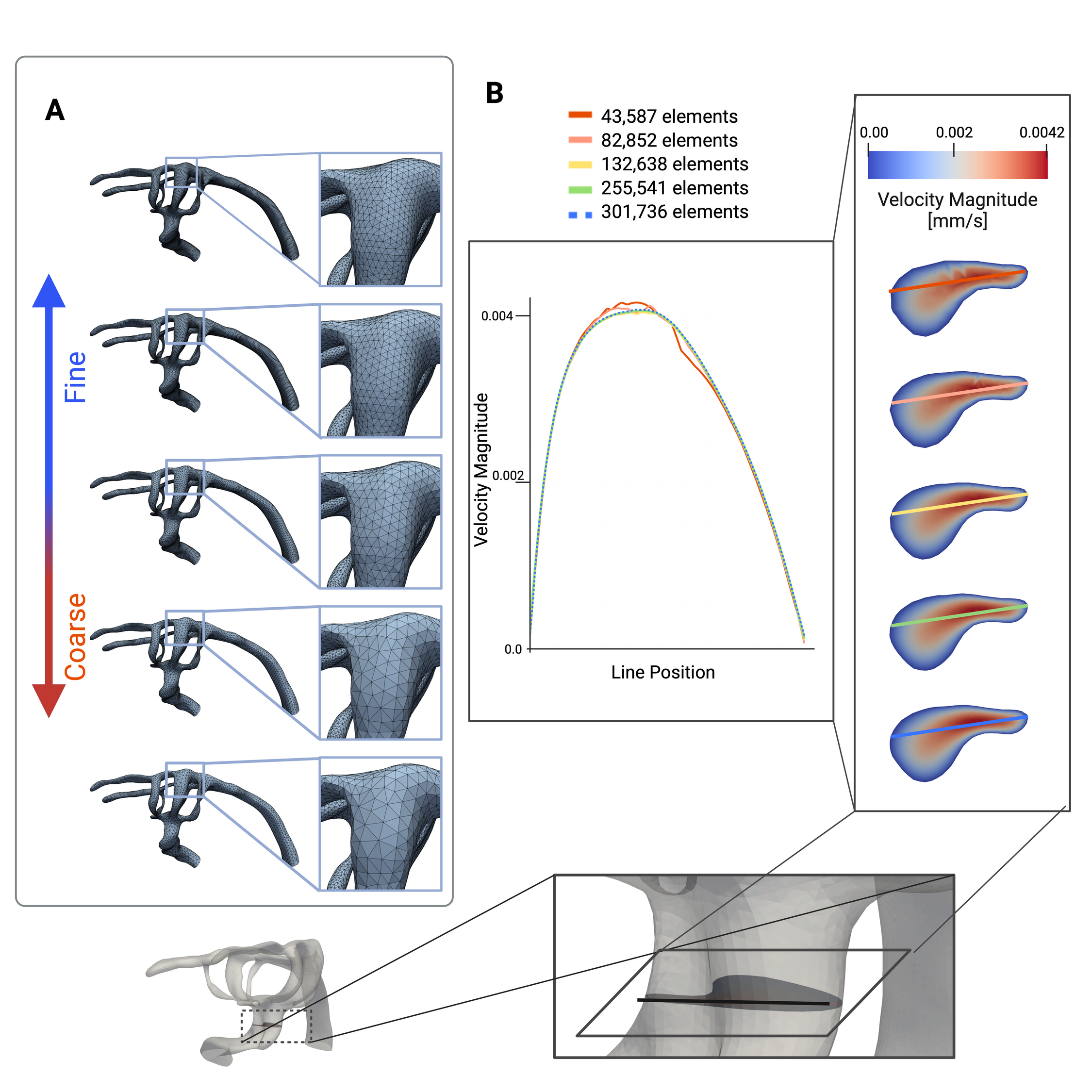

Supplement: S3 Fig — (A) We constructed increasingly fine meshes of a template embryo ranging from 43,587 tetrahedral elements to 301,736 elements. (B) We examined simulation results under these different levels of mesh refinement by sampling velocity magnitude across a line drawn across the lumen shown above. Cross sections of the lumen with the simulations under the different mesh refinements are also shown with the lines that were used to sample the velocity. (TIF) [file pone.0322233.s003.tif]
