# Supplementary material for: An anatomically informed computational fluid dynamics modeling approach for quantifying hemodynamics in the developing heart
Source: PLoS One. 2025 May 19;20(5):e0322233. doi: 10.1371/journal.pone.0322233 (PMC12088024; doi:10.1371/journal.pone.0322233)
Supplement: Attachment — Submitted filename: Reviewer_Response_PLOS_ONE.docx [file pone.0322233.s005.docx]

We thank the reviewers for their thoughtful and thorough critique of our manuscript. Several important points were raised by the reviewers, and by addressing these topics we feel that the manuscript has been substantially improved. Please find a point-by-point response to the reviewers concerns below:

**Reviewer One:**

**1.The computational pipeline is applied to chick embryos. How can the findings and methodology be translated to humans? This includes not only the procedural aspects but also the implications of the findings, which remain unclear.**

Thank you for bringing up this important point. The potential translational impact of this protocol to human health and human heart development was the central motivating factor for undertaking this work.

The chick is a classical model of four chambered heart development which is similar in size, structure, and function to the human heart across the stages that we have examined in this manuscript (1–3). As such, the cardiac development field frequently uses the chick as an experimental model for determining how hemodynamic perturbations influence normal four chambered morphogenesis.

In addition, previous studies have begun to move away from using “idealized” embryonic cardiac geometries to run computational modeling with the intent of determine how specific anatomical variations influence flow patterns, pressures, and wall shear stresses that drive the formation of congenital heart defects. Here, we have designed the pipeline described in this manuscript to rapidly attain whole luminal geometries from large numbers of samples, translate and scale these geometries for comparative morphometric analysis, and translate these geometries for CFD modeling. While this is currently tailored to the chick embryonic system, we feel that as fetal ultrasound modalities continue to improve (4), image classification, morphometric analysis, and computational modeling techniques can become powerful diagnostic metrics for determining the likelihood and potential severity of heart defects well-before they emerge in utero.

We add clarifications of the implications of this study in several sections throughout the manuscript including in the introduction on line 71:

Ultimately, this fast pipeline for comparing morphological features and variation to hemodynamic patterns in the embryonic chick heart model can be extended to other model systems in order to predict how differences in individual morphology and hemodynamics can impact heart development. Additionally, it is fully expected that computational modeling approaches can be used in the future to investigate hemodynamics in human heart development, as fetal cardiac imaging improves (4). Given the similarities in size, structure, and function between human and chick heart development, the chick serves as an ideal model system to run proof-of-principle analysis for such techniques.

Additionally, we address how GMM and CFD models can be used together to be applied to human heart development in line 387:

This pairing of GMM with CFD models could also be applied to predicting critical morphological differences that lead to altered blood flow dynamics and impact human heart development. As imaging for fetal hearts rapidly improves, having a detailed understanding and robust method for analyzing this relationship between morphology and hemodynamics already established would be an extremely useful tool.

On line 489 we reiterate how these findings can be applied to human heart health:

Our findings contribute to the growing body of literature on CFD models of embryonic chick AAs by providing a fast and cheap imaging method that can easily be used to generate cell-accurate geometries. Ultimately the CFD and morphometric analysis can be extended to compare hemodynamic and morphological features in several ways, including overlaying gene expression patterns with flow sensitive regions. Methods for comparing flow dynamics within individual morphologies will furthermore allow for translations to human fetal heart analysis.

**2.Regarding the numerical simulations, it is stated that a laminar flow model is considered. Does this imply that no turbulence model is employed? However, would a turbulence model be truly necessary given the nature of blood flow and the resolution of the computational mesh?**

The reviewer raises an important point that we have clarified in the manuscript (see below). To directly answer the reviewer, flow in this system is laminar. Turbulent flow would occur when inertial forces dominate over viscous forces. The Reynolds number, which is a nondimensional number describing the ratio of inertial forces to viscous forces, can be used to characterize flow as laminar or turbulent. Generally, if the Reynolds number is below 2000, then flow is laminar, and turbulence will not occur. Several previous *in vivo* studies have demonstrated that Reynolds numbers at these stages of development are orders of magnitude lower than would be required for turbulent flow. For instance, Midgett et al. demonstrate that the Reynolds number is less than 9 in the outflow tract at HH18, using doppler Optical Coherence Tomography (5). Kowalski et al. review previous *in vivo* studies that have shown the Reynolds number is between 0.1 and 9 in the embryonic chick’s cardiovascular system at the stages of our model (6), indicating that flow is laminar. Additionally, CFD studies modeling blood flow at this stage in the outflow tract and AAs also describe blood flow as laminar (7,8). Therefore, we did not model turbulent flow. We did, however, focus on pulsatile laminar flow, rather than steady flow.

We have clarified these points in the manuscript on line 308:

Previous studies have calculated Reynolds numbers to be between 0.1 to 9.0 for the stages of development we have analyzed (1,3,8,9)(1,2,3,4). This would indicate that intracardiac blood flow is highly laminar during early cardiac morphogenesis (6,7,10).

We reiterate this when discussing our results on line 327:

This is consistent with laminar flow and previous reports of laminar flow in CFD models of this region (7).

On line 347:

Since the location of peak velocity streamlines is maintained across different flow phases, this further supports the finding that there is little convective mixing and that flow is laminar.

On line 362:

This maintenance of neighbors of streamline sets from the outflow tract to the dorsal aorta was consistent across embryos (Fig 6G) and is also consistent with laminar flow.

**3.Unsteady flow is considered in a fixed computational domain. Is it correct?**

Yes, that is correct. We employ the full Navier Stokes equations which account for unsteady flow. We model pulsatile pressure at the inflow and outflow boundaries, so there is not a fixed pressure and the resulting flow is pulsatile.

**4.The inflow and outflow boundary conditions are briefly mentioned, but their implementation lacks clarity. Given that the flow is highly sensitive to these boundary conditions, the numerical results—particularly the mean velocity and wall shear stress—can be significantly influenced by their choice. How robust are the results and, consequently, the study’s findings under these assumptions?**

Thank you for bringing up these salient points. A quantitative sensitivity analysis perturbing the inflow and outflow boundary conditions or other parameters such as viscosity could be used to understand how delicate the resulting WSS and velocity of the model are to these input conditions. Additionally, an uncertainty quantification could be used to indicate a range of probable output values, based on the input parameters, rather than a single output value.

We agree with the reviewer that the model could benefit from a quantitative sensitivity analysis and uncertainty quantification. Unfortunately, these analyses are far beyond the scope of the current study. Indeed, given the complexity of these types of analysis, the standard in the field has been to not include an uncertainty quantification in the initial model implementation. In fact, a 2021 review article on computational approaches to modeling embryonic heart hemodynamics (11) reports that most CFD models of the cardiovascular system do not consider the uncertainty of the input parameters or the sensitivity of the output values to them (11). However, we think it would be excellent to conduct a quantitative sensitivity analysis and uncertainty quantification in future studies and developments of the current model. Indeed, we feel the model we have implemented is ideal for this type of analysis.

Several uncertainty quantification techniques and sensitivity analyses have been implemented for some CFD models of the cardiovascular system. Computational studies that perform these comprehensive uncertainty quantifications and global sensitivity analyses typically draw from and expand previously implemented models or use idealized models. The main results from these papers are focused on the sensitivity of parameters rather than the clinical or mechanistic implications of the model results (12–14). Uncertainty quantification methods are rooted in probability theory, where probability distribution functions are used as input values and based on empirical information on the input values (12). Output values are then represented as distributions based on the model’s inputs (12). There are a variety of methods we could use to implement an uncertainty quantification that have advantages and drawbacks (11). For instance, Monte Carlo methods can been used for uncertainty quantification in CFD simulations, but they are costly computationally (12,13). The generalized polynomial chaos expansion method is an alternative uncertainty quantification method that has gained traction recently in the field and is more computationally efficient than the Monte Carlo method (12,13).

In fact, we have designed this pipeline to be amenable to these comparisons and perturbations. Indeed, it is our plan to use the generalized polynomial chaos expansion method to calculate the uncertainty quantification of our output in a future study. Additionally, we plan to implement the Sobol indices to determine input parameter sensitivity as has been previously done (12). However, these analyses would be quite extensive and are probably not appropriate in the context of the current manuscript.

Given the above, we have included this limitation in our discussion on line 424:

Our model currently does not include uncertainty quantification of the results or sensitivity analysis of model parameters. Although CFD studies on the cardiovascular system do not routinely report sensitivity analysis (11), several groups have implemented these analyses for CFD studies of both the mature and developing cardiovascular system (12–15). In future studies, we would like to examine the sensitivity of input parameters such as the inflow and outflow boundary conditions and the blood viscosity. Additionally, we would also like to conduct an uncertainty quantification such as the generalized polynomial chaos expansion method which has been previously implemented in CFD uncertainty quantification papers to provide a more comprehensive description of probable output values (11,12).

**5.Geometrical imprecision in the geometrical model can significantly impact the results of numerical simulations. How do such inaccuracies affect the robustness and reliability of the findings? Has this been quantified or accounted for in the analysis?**

We strongly agree with the reviewer that the model geometry has the potential to impact the results. In fact, Table 1 in our manuscript highlights that differences in reported model geometry of the same system and development stage may greatly impact the simulated hemodynamic results. Additionally, previous studies have stated that model geometry is the most challenging feature to obtain for embryonic heart CFD studies but geometry is also the most important feature for obtaining accurate results in these studies (2,16).

Expanding the sensitivity analysis and uncertainty quantification described in the previous response to perturb the geometry of the model could address how the geometry impacts the output. We expect that the system that we have constructed in the manuscript would be ideal for running this type of robust sensitivity analysis and think this is a major strength to the pipeline we have designed. The system is suitable for comparing several geometries and their CFD results as well as their morphometrics, simultaneously. However, a complete description and implementation of such a sensitivity analysis would likely require an entirely separate manuscript. We would stress, that our goals in the current manuscript were to describe the construction of our pipeline and we feel there are many downstream utilities.

We are actually quite excited to pursue geometry uncertainty quantification, especially as imaging methods improve. The GMM analysis we have conducted in this study could serve to identify which morphological features vary the most. These features could be the first to be perturbed in the uncertainty quantification and sensitivity analysis. In addition to geometric features identified through the GMM analysis, we could also perturb morphological features such as individual AA length and diameter for each pair of AAs. We have constructed our platform to be amenable to such a sensitivity analysis study and could easily alter the geometry to perform these studies. We state this sentiment on line 465:

In addition to conducting a sensitivity analysis on physiological model parameters such as inflow and outflow boundary conditions, we would extend the analysis to examine the impact of variations of model geometry on output. The GMM analysis could be used as an unbiased but directed method to identify which morphological features vary the most, and these features could be included in the sensitivity analysis.

**Reviewer Two:**

**Similar work - using slightly different tools - has been implemented by several groups, including the Butcher group at Cornell, and Pekkan and Keller groups. Light sheet microscopy has also been implemented in the living zebra fish embryo heart - and CFD models (with moving walls - more advanced than the ones by the author) have been developed by Marsden at Stanford and Yap in the UK. 4D reconstructions of the heart have also been achieved using OCT imaging by several groups, including groups at Baylor and Case Western, and wall shear stresses have been estimated from measured flow and through CFD of the moving heart. All these works are grossly minimized or neglected by the authors, who point to "lack of protocols" - and who claim their methods are superior, as nobody could before calculate flow or wall shear stress. In fact their Table 1 omits several works.**

We strongly agree with the reviewer that several groups have generated excellent CFD models that have been implemented to study cardiac development. We are alarmed if we gave the impression of diminishing this previous work in the field and we would emphasize that we did not explicitly state, nor did we intend to imply that our methods are “superior” or that “nobody could before calculate flow or wall shear stress.” Rather, we compared previously calculated wall shear stress values in Table 1. Our purpose in presenting this study is to provide a detailed description or data acquisition, translation, analysis, and computational modeling. As stated above in our response to reviewer 1, we feel that the pipeline we describe in this manuscript is ideally suited for a broad variety of downstream hemodynamic analysis in the context of morphological variation.

We’d like to address all parts of this comment, beginning with the following:

**Similar work - using slightly different tools - has been implemented by several groups, including the Butcher group at Cornell, and Pekkan and Keller groups. …All these works are grossly minimized or neglected by the authors, who point to "lack of protocols" - and who claim their methods are superior, as nobody could before calculate flow or wall shear stress. In fact their Table 1 omits several works.**

We agree with the reviewer that excellent work has been carried out by the groups mentioned. We have already acknowledged multiple publications by these groups several times in the manuscript. Our initial submission cited 5 manuscripts produced by the Butcher group and 4 publications authored by Pekkan and/or Keller. In the original manuscript these groups are listed as references 7,8,24,27,65, and 29-32, respectively. Below we have provided several of these citations indicated on the line as they appeared in the original manuscript as well their location in the resubmitted manuscript.

The Butcher group is cited in the following lines in the original manuscripts (the location in the revised manuscript is shown in parenthesis):

In the original manuscript, the citation appears in line 59 (line 57):

“Indeed, phenotypes mimicking human CHDs can be robustly reproduced by cardiac outflow tract banding or vasculature ligation in embryonic model organisms(17,18), suggesting perturbed hemodynamics contribute to abnormal cardiovascular development and function.”

The citation appeared in line 62 on the original manuscript (line 60):

“Particularly, these surgical studies have shown that wall shear stress (WSS) regulates several aspects of cardiogenesis through mechanosensitive pathways(19,20).”

Citation appears in line 114 in the original manuscript (line 121):

“Chick embryos are attractive experimental models for studying normal and pathological blood flow in cardiogenesis(21–27) due to their similar order of developmental events(23,28)”

Citation appears in line 116 ( rephrased on line 128):

“Embryonic chick hearts have previously been modeled in CFD studies(8,21,29,30);”

Citation appears in line 121 (line 134):

“Unfortunately, this level of detail is often lost in subject-averaged models(8)”

Citation appears in line 415 (line 453):

“Additionally, this cohort-based approach revealed elliptical cross sections and aortic arch diameters on the same order of magnitude as AAs imaged in other cohort-based studies(8,31).”

The Keller and/or Pekkan group is cited in the following sentences:

Citation appears in line 116 and 118 in the following sentence (line 128 rephrased):

“Embryonic chick hearts have previously been modeled in CFD studies(8,21,29,30); however, most of these intracardiac hemodynamics studies employ idealized geometries or combine several individual anatomies to form a single representative geometry of a developing chick heart(7,32).”

Citation appears in line 132 (line 146):

“The AAs undergo significant remodeling during embryonic growth and contribute to the aorta and pulmonary artery [(29)].”

Citation appears in line 186 (line 200):

“Typically, imaging the AAs is difficult due to their complicated geometry, small size, and dorsal/anterior position relative to the looping heart, so alternative methods such as ink injections have been used to map the arches(32).”

Citation appears in line 268 (line 282):

“This pattern of AA IV arch pair having the smallest average diameter and AA III having the largest average diameter is consistent with previous reports among the diameters of these three arch pairs measured at similar stages[31].”

Citation appears in line 392 (line 419):

“This study, as well as most CFD studies of these stages and later in embryonic chick development, models blood as a Newtonian fluid(32,33).”

In addition, we cite these authors in Table 1. We did limit the contents of Table 1 to various imaging methods that have been used to generate CFD models of the aortic arches during chick heart development. The table is not meant to serve as an exhaustive list of all CFD models of various embryonic hearts in different model systems as this would be more appropriate for a review paper. We have stated this in the manuscript on line 136:

“as average aortic arch (AA) midpoint diameter. These studies also report a broad range of shear stress patterns, and we have summarized a non-exhaustive list of examples of reported geometric values and WSS ranges that different imaging techniques of embryonic chick AA CFD models have yielded (Table 1).”

The purpose of Table 1 is to illustrate that the WSS values derived from these CFD models may be directly related to the imaging methods used to generate the computational domains of these models, as these models all reported different geometric dimensions and WSS values in the AAs. Here again, we would emphasize that we do not claim anywhere in the manuscript that flow and WSS have yet to be calculated by these groups. Rather, we reference the calculated WSS these groups have reported.

We have attempted to clarify this and to put our approach in better context on line 476:

CFD modeling of this system is not novel in-and-of itself, however, we have attempted to develop methods that are fast and easily customizable for a broad variety of hemodynamic studies across specimen-specific morphologies. This pipeline can easily be modified and extended to explore various aspects of cardiovascular development in embryonic chicks and other model organisms and to conduct robust sensitivity analysis on flow parameters and model geometry across individual morphologies.

We would also like to address the “lack of protocols” term Reviewer 2 refers to:

We do state it the manuscript that there has been a “lack of protocols in generating cell-accurate descriptions of the embryonic cardiovascular anatomy through which to run simulations”. Our criterion for generating these geometries was the ability to resolve individual cells both within the heart and lining the vasculature. The fluorescence light sheet microscopy imaging technique employed here achieves this.

**Light sheet microscopy has also been implemented in the living zebra fish embryo heart - and CFD models (with moving walls - more advanced than the ones by the author) have been developed by Marsden at Stanford and Yap in the UK. 4D reconstructions of the heart have also been achieved using OCT imaging by several groups, including groups at Baylor and Case Western, and wall shear stresses have been estimated from measured flow and through CFD of the moving heart.**

We agree with the reviewer that LSFM has been implemented to study hemodynamics in the zebrafish. We also referenced this in the original manuscript on line 380:

“LSFM has already been used to visualize shear stress-activated signaling pathways, such as Notch signaling in embryonic zebrafish hearts(34).”

We have updated the manuscript to include the Marsden group’s use of LSFM for a CFD study in the zebrafish in line 177 in the revised manuscript:

While light sheet microscopy has been proposed for use and implemented in CFD studies in zebrafish hearts (35,36), currently it is not routinely implemented for this purpose in four-chambered hearts.

We did not include these studies in Table 1 as they were performed in two-chambered hearts. We did add citations of studies of WSS in heart development focusing on other model organisms such as the zebrafish and mouse beginning on line 57:

Indeed, phenotypes mimicking human CHDs can be robustly reproduced by cardiac outflow tract banding or vasculature ligation in embryonic model organisms [6–9], suggesting perturbed hemodynamics contribute to abnormal cardiovascular development and function. Particularly, studies have shown that wall shear stress (WSS) regulates several aspects of cardiogenesis through mechanosensitive pathways [10–14].

We limit the scope of this study to focus on four-chambered heart model organisms. In total, the findings from CFD studies of chicks, and other model organisms with four-chambered hearts can clarify how the hemodynamic environmental contributes to heart development and defects in humans. We reiterate our choices for using the four-chambered animal model, LSFM, and CFD. We clarify how using LSFM for the application can ultimately elucidate heart development in humans starting on line 406:

Ultimately, LSFM based imaging methods to inform the computational domain can also be paired with other methods, such as fluorescent RNA *in situ* hybridization (37,38) and/or immunofluorescence, to directly compare mechanosensitive gene/protein expression with the CFD simulations. In this respect, LSFM is an extremely flexible and powerful system generating geometries for computational analysis.

We reiterate the choice for using a four-chambered heart animal model system on line 470:

It is well established that hemodynamics plays a critical role in shaping the course of a developing heart in humans as well as other organisms with four-chambered hearts.

We admire the outstanding OCT work of the groups at Baylor and Case Western. However, these groups do not use the OCT imaging to develop CFD models, which is why the manuscript does not highlight them in Table 1.

**CFD models employed by the authors are not validated and meshes employed are not rigorously tested through a convergence study. Moreover, the geometry employed and boundary conditions do not seem appropriate. CFD boundary conditions are not shown - and comparisons with actual velocity measurements are not used to validate the model results.**

We agree with the reviewer that this is an important consideration. We conducted a convergence mesh study during our initial model implementations. We have now included this as a supplementary figure, S3 (See below). Meshes were refined such that every mesh consisted of at least 100,000 tetrahedral elements since little difference in flow results after this level of refinement (See Fig S3 B).

We have included a convergence study figure, in which we construct increasingly fine meshes ranging from 43,587 elements to 301,736 elements (Fig S3 A). To examine how mesh size influenced the numerical results, we compared velocity profiles across the different mesh refinements. From S3B, the numerical results converge in meshes of 132,638 or more elements. We used the model containing 255,541 elements to conduct the simulations. We applied this level of refinement to all specimen models.

**Fig S3. Mesh convergence study.** (A) We constructed increasingly fine meshes of a template embryo ranging from 43,587 tetrahedral elements to 301,736 elements. (B) We examined simulation results under these different levels of mesh refinement by sampling velocity magnitude across a line drawn across the lumen shown above. Cross sections of the lumen with the simulations under the different mesh refinements are also shown with the lines that were used to sample the velocity.

We placed the Fig. S3 caption below line 218 and included a sentence referencing Fig. S3 on line 218:

We conducted a mesh convergence study (Fig. S3).

We use zero slip boundary conditions and impose pulsatile pressure boundary conditions that we interpolated form previously published pressure readings from (39). This was stated in the original manuscript on line 292:

“No slip boundary conditions were imposed on intracardiac and vessel walls, which are assumed to be rigid and impermeable. Sinusoidal pulsatile pressure profiles, determined by digitizing previously reported pressure profiles in the ventricle and dorsal aorta [61], were imposed at the inflow and outflow boundaries, respectively.”

We do agree that velocity measurements would be an excellent validation for this study to incorporate. We did not focus on this in the current manuscript as our data is consistent with ranges of WSS reported by with previous studies (8,32). We now reference this on line 330:

The ranges of WSS we observed are similar and overlap with previous WSS ranges reported (10,32).

**The authors start talking about the heart and flow in the heart - but end up modeling only the heart outflow tract and the extracardiac aortic arches and dorsal aorta. It would be more appropriate to neglect the heart outflow tract portion, as it is wrongly captured from fixed embryos (see below). In the authors' models, it would make more sense to start with the aortic sac, then model the conection to the arches and then the aorta. All these are relatively 'fix' portions of vasculature in the embryos.

The outflow tract is a very dynamic cardiac structure: it opens and closes during the cardiac cycle as blood flows through it. But instead of modeling this motion, the authors chose a static (fix) geometry. While this might be a fair approximation (and certainly employed before from CT images) - the procedures employed by the authors to fix the embryos do not seem to arrest the heart properly. The authors mention "the degree of stenosis in the outflow tract" that is apparently different among samples. This likely reflects the fact that the outflow tract of different embryos is arrested at different points in the cardiac cycle (e.g. open outflow tract, semi-closed outflow tract) given the impression that sometimes the outflow tract has "stenosis". There is no "stenosis" in a normal outflow tract - what the authors observed are the outflow tract cushions that are more or less obvious depending on how close or open the outflow tract walls are. The authors should be aware of anatomy and motion of the outflow tract and its implications before attempting to model it. In their models, it would be simpler to neglect the outflow tract.**

We agree with the reviewer that the OFT is not static. Indeed, we have begun the construction of fluid structure interaction models that incorporates the movement of the embryonic atria, atrioventricular junction, ventricles, and OFT into our examinations of early cardiac hemodynamics. These models are based on FSI modeling techniques that we have recently published for the whole adult heart (40). Ultimately, we feel that incorporating motion (not just of the OFT, but also of the upstream chambers and cushions) will provide substantial utility. However, the FSI models that we are currently constructing are well beyond the scope of the current manuscript. We would also emphasize that previous CFD studies conducted at these stages also model AA flows using a static OFT (7,32). We have included the OFT in our modeling as we felt its morphology would impact flow through the AA, however, we have now acknowledged this as a limitation on line 444:

However, we do recognize that a current limitation of this study is that the model is static, including the OFT. Although the AAs are relativity stiff, *in vivo*, the OFT is relatively dynamic. In future studies we would like to model the OFT to be dynamic, however that is outside the scope of the study.

We would also apologize that our description of fixation as described in the methods was incomplete. We did in fact arrest cardiac motion prior to fixation using 2,3 butanedione monoxide (BDM). Embryos were isolated in hanks buffered saline solution containing 20mM BDM for ten minutes (which arrested all motion) and then transferred into 6-well plates containing ice cold 4% PFA. This has been corrected beginning on line 496:

Following the described micro-injections, the embryos were removed from the eggs and isolated in Hanks Buffered Saline Solution containing 20 mM 2,3 butanedione monoxide for ten minutes, which arrested cardiac motion. Embryos were then transferred into 6-well plates containing ice cold 4% PFA. The embryos were shaken in PFA overnight at 4°C prior to clearing.

**Authors also mention that vessels have an elliptical cross-sections - how do they know this is not an artifact from the embryo fixation, and/or imaging?**

This is an interesting point. We assume that the result of elliptical cross sections in the AAs is not an artifact from fixing, as our imaging revealed circular cross sectional areas in other embryonic vessels of similar size in similar tissue beds (see image below). We feel that it would be highly unlikely that only the AAs would be distorted by fixation artifacts. In the figure, we have compared the ratios of a pair of AAs to the ratios of cranial arteries in the head of an E4 embryo cleared using the same protocol. The ratios indicate that the AAs are more elliptical than the cranial AAs. This supports our assumption that the reported elliptical structure of the AAs is not an artifact from imaging or the fixation method we used.

**Later in the paper, the authors are then "surprised" to find that regions of high flow velocities also corresponds to regions of high wall shear stress, and that inertia effects are negligible. This is simply due to the low flow Reynolds number. This is not surprising given the small size of the embryo and its heart adn vasculature.**

Yes, we agree that regions of high flow velocity will correspond to high wall shear stress. Additionally, we agree that the Reynolds number in this flow is extremely low and that flow is dominated by viscous effects. We apologize for any confusion, but we had used the term “surprisingly” in reference to the finding that peak flow and WSS occurred in the central AA in the model. In fact, in the original manuscript Figure 5 is entitled:

“**Fig 5.** **Blood flow velocity streamlines are consistent with WSS patterns**.”

And in Figure 5(A) in the original manuscript, we had stated our expectation of peak WSS to correspond to peak velocity:

(A) Blood flow velocity streamlines are observed during peak and accelerating flow, exhibiting similar temporal and spatial patterns compared to WSS, consistent with the finding that there is low convective flow**. As expected, blood flow velocity streamlines indicate elevated flow in the same regions as elevated WSS across variations in embryonic anatomies.**

We regret if this was interpreted as surprise at basic parameters of the system. Given the reviewers comments we have rephrased the section beginning on line 333 to clarify our word choice:

This allowed us to determine that the central pair of AAs (AA III) displayed the highest average WSS across our samples (Fig 4G). AA III was the shortest pair present in our embryos but was not the most stenosed arch pair (Fig 4D-F). Of note, each AA pair experienced similar pressure during the cardiac cycle, despite the central pair undergoing higher WSS (Fig 4F, G).

Additionally, we rephrase our results beginning on line 439:

Our findings reveal that the AAs are exposed to different wall shear stresses during this stage of development. Radii alone in the AAs was not predictive of peak WSS or blood flow velocity within.

References for Response:

1.	Wittig JG, Münsterberg A. The Chicken as a Model Organism to Study Heart Development. Cold Spring Harb Perspect Biol. 2020 Aug;12(8):a037218.

2.	Yalcin HC, Shekhar A, McQuinn TC, Butcher JT. Hemodynamic Patterning of the Avian Atrioventricular Valve. Dev Dyn. 2011 Jan;240(1):23–35.

3.	Jensen B, Chang YH, Bamforth SD, Mohun T, Sedmera D, Bartos M, et al. The changing morphology of the ventricular walls of mouse and human with increasing gestation. Journal of Anatomy. 2024;244(6):1040–53.

4.	Barber N, Freud L. Advances in Fetal Cardiac Imaging and Intervention. CJC Pediatric and Congenital Heart Disease. 2024 Feb 1;3(1):33–42.

5.	Midgett M, Goenezen S, Rugonyi S. Blood flow dynamics reflect degree of outflow tract banding in Hamburger–Hamilton stage 18 chicken embryos. J R Soc Interface. 2014 Nov 6;11(100):20140643.

6.	Kowalski WJ, Pekkan K, Tinney JP, Keller BB. Investigating developmental cardiovascular biomechanics and the origins of congenital heart defects. Front Physiol [Internet]. 2014 Oct 21 [cited 2025 Feb 5];5. Available from: https://www.frontiersin.org/journals/physiology/articles/10.3389/fphys.2014.00408/full

7.	Kowalski WJ, Dur O, Wang Y, Patrick MJ, Tinney JP, Keller BB, et al. Critical Transitions in Early Embryonic Aortic Arch Patterning and Hemodynamics. PLOS ONE. 2013 Mar 21;8(3):e60271.

8.	Lindsey SE, Butcher JT, Vignon-Clementel IE. Cohort-based multiscale analysis of hemodynamic-driven growth and remodeling of the embryonic pharyngeal arch arteries. Development. 2018 Oct 15;145(20):dev162578.

9.	Sedmera D, Pexieder T, Vuillemin M, Thompson RP, Anderson RH. Developmental patterning of the myocardium. Anat Rec. 2000 Apr 1;258(4):319–37.

10.	Hogers B, DeRuiter M c., Baasten A m. j., Gittenberger-de Groot A c., Poelmann R e. Intracardiac Blood Flow Patterns Related to the Yolk Sac Circulation of the Chick Embryo. Circulation Research. 1995 May;76(5):871–7.

11.	Salman HE, Yalcin HC. Computational Modeling of Blood Flow Hemodynamics for Biomechanical Investigation of Cardiac Development and Disease. Journal of Cardiovascular Development and Disease. 2021 Feb;8(2):14.

12.	Waldrop LD, He Y, Battista NA, Neary Peterman T, Miller LA. Uncertainty quantification reveals the physical constraints on pumping by peristaltic hearts. Journal of The Royal Society Interface. 2020 Sep 9;17(170):20200232.

13.	Ho NN, Lee KY, Lee SW. Uncertainty quantification of computational fluid dynamics-based predictions for fractional flow reserve and wall shear stress of the idealized stenotic coronary. Front Cardiovasc Med [Internet]. 2023 Nov 27 [cited 2025 Feb 8];10. Available from: https://www.frontiersin.org/journals/cardiovascular-medicine/articles/10.3389/fcvm.2023.1164345/full

14.	Sankaran S, Kim HJ, Choi G, Taylor CA. Uncertainty quantification in coronary blood flow simulations: Impact of geometry, boundary conditions and blood viscosity. Journal of Biomechanics. 2016 Aug 16;49(12):2540–7.

15.	Fleeter CM, Geraci G, Schiavazzi DE, Kahn AM, Marsden AL. Multilevel and multifidelity uncertainty quantification for cardiovascular hemodynamics. Computer Methods in Applied Mechanics and Engineering. 2020 Jun 15;365:113030.

16.	Birchall D, Zaman A, Hacker J, Davies G, Mendelow D. Analysis of haemodynamic disturbance in the atherosclerotic carotid artery using computational fluid dynamics. Eur Radiol. 2006 May 1;16(5):1074–83.

17.	Midgett M, Rugonyi S. Congenital heart malformations induced by hemodynamic altering surgical interventions. Front Physiol. 2014;5:287.

18.	Salman HE, Alser M, Shekhar A, Gould RA, Benslimane FM, Butcher JT, et al. Effect of left atrial ligation-driven altered inflow hemodynamics on embryonic heart development: clues for prenatal progression of hypoplastic left heart syndrome. Biomech Model Mechanobiol. 2021 Apr 1;20(2):733–50.

19.	Groenendijk BCW, Van der Heiden K, Hierck BP, Poelmann RE. The Role of Shear Stress on ET-1, KLF2, and NOS-3 Expression in the Developing Cardiovascular System of Chicken Embryos in a Venous Ligation Model. Physiology. 2007 Dec;22(6):380–9.

20.	Wang M, Lin BY, Sun S, Dai C, Long F, Butcher JT. Shear and hydrostatic stress regulate fetal heart valve remodeling through YAP-mediated mechanotransduction. Yutzey K, Stainier DY, Vermot J, editors. eLife. 2023 Apr 20;12:e83209.

21.	Courchaine K, Gray MJ, Beel K, Thornburg K, Rugonyi S. 4-D computational modeling of cardiac outflow tract hemodynamics over looping developmental stages in chicken embryos. Journal of cardiovascular development and disease. 2019;6(1):11.

22.	Menon V, Eberth JF, Goodwin RL, Potts JD. Altered hemodynamics in the embryonic heart affects outflow valve development. Journal of cardiovascular development and disease. 2015;2(2):108–24.

23.	Alser M, Shurbaji S, Yalcin HC. Mechanosensitive Pathways in Heart Development: Findings from Chick Embryo Studies. Journal of Cardiovascular Development and Disease. 2021;8(4):32.

24.	Midgett M, Thornburg K, Rugonyi S. Blood flow patterns underlie developmental heart defects. American Journal of Physiology-Heart and Circulatory Physiology. 2017;312(3):H632–42.

25.	Midgett M, López CS, David L, Maloyan A, Rugonyi S. Increased hemodynamic load in early embryonic stages alters endocardial to mesenchymal transition. Frontiers in physiology. 2017;8:56.

26.	Hogers B, DeRuiter MC, Gittenberger-de Groot AC, Poelmann RE. Extraembryonic venous obstructions lead to cardiovascular malformations and can be embryolethal. Cardiovascular Research. 1999;41(1):87–99.

27.	Hogers B, DeRuiter M, Gittenberger-de Groot A, Poelmann R. Unilateral vitelline vein ligation alters intracardiac blood flow patterns and morphogenesis in the chick embryo. Circulation research. 1997;80(4):473–81.

28.	Lindsey SE, Butcher JT, Yalcin HC. Mechanical regulation of cardiac development. Frontiers in physiology. 2014;5:318.

29.	Lindsey SE, Menon PG, Kowalski WJ, Shekhar A, Yalcin HC, Nishimura N, et al. Growth and hemodynamics after early embryonic aortic arch occlusion. Biomech Model Mechanobiol. 2015 Aug;14(4):735–51.

30.	Kowalski WJ, Teslovich NC, Dur O, Keller BB, Pekkan K. Computational hemodynamic optimization predicts dominant aortic arch selection is driven by embryonic outflow tract orientation in the chick embryo. Biomechanics and modeling in mechanobiology. 2012;11(7):1057–73.

31.	Lindsey SE, Vignon-Clementel IE, Butcher JT. Assessing Early Cardiac Outflow Tract Adaptive Responses Through Combined Experimental-Computational Manipulations. Ann Biomed Eng. 2021 Dec 1;49(12):3227–42.

32.	Wang Y, Dur O, Patrick MJ, Tinney JP, Tobita K, Keller BB, et al. Aortic Arch Morphogenesis and Flow Modeling in the Chick Embryo. Ann Biomed Eng. 2009 Jun 1;37(6):1069–81.

33.	Ho S, Chan WX, Phan-Thien N, Yap CH. Organ Dynamics and Hemodynamic of the Whole HH25 Avian Embryonic Heart, Revealed by Ultrasound Biomicroscopy, Boundary Tracking, and Flow Simulations. Sci Rep. 2019 Dec 2;9:18072.

34.	Messerschmidt V, Bailey Z, Baek KI, Bryant R, Li R, Hsiai TK, et al. Light-sheet Fluorescence Microscopy to Capture 4-Dimensional Images of the Effects of Modulating Shear Stress on the Developing Zebrafish Heart. J Vis Exp. 2018 Aug 10;(138):57763.

35.	Fei P, Lee J, Packard RRS, Sereti KI, Xu H, Ma J, et al. Cardiac Light-Sheet Fluorescent Microscopy for Multi-Scale and Rapid Imaging of Architecture and Function. Sci Rep. 2016 Mar 3;6(1):22489.

36.	Vedula V, Lee J, Xu H, Kuo CCJ, Hsiai TK, Marsden AL. A method to quantify mechanobiologic forces during zebrafish cardiac development using 4-D light sheet imaging and computational modeling. PLOS Computational Biology. 2017 Oct 30;13(10):e1005828.

37.	Wang F, Flanagan J, Su N, Wang LC, Bui S, Nielson A, et al. RNAscope. J Mol Diagn. 2012 Jan;14(1):22–9.

38.	Kanatani S, Kreutzmann JC, Li Y, West Z, Larsen LL, Nikou DV, et al. Whole-brain spatial transcriptional analysis at cellular resolution. Science. 2024 Nov 22;386(6724):907–15.

39.	Shi L, Goenezen S, Haller S, Hinds MT, Thornburg KL, Rugonyi S. Alterations in pulse wave propagation reflect the degree of outflow tract banding in HH18 chicken embryos. Am J Physiol Heart Circ Physiol. 2013 Aug 1;305(3):H386-396.

40.	Davey M, Puelz C, Rossi S, Smith MA, Wells DR, Sturgeon GM, et al. Simulating cardiac fluid dynamics in the human heart. PNAS Nexus. 2024 Oct;3(10):pgae392.
